# Principles of using Cold Atmospheric Plasma Stimulated Media for Cancer Treatment

**DOI:** 10.1038/srep18339

**Published:** 2015-12-17

**Authors:** Dayun Yan, Annie Talbot, Niki Nourmohammadi, Xiaoqian Cheng, Jerome Canady, Jonathan Sherman, Michael Keidar

**Affiliations:** 1 Department of Mechanical and Aerospace Engineering, The George Washington University, Science & Engineering Hall, 800 22nd Street, NW, Room 3550, Washington, DC 20052, USA; 2 Columbian College of Arts and Sciences, The George Washington University, Phillips Hall, 801 22nd Street, NW, Suite 212, Washington, DC 20052, USA; 3 Department of Biological Sciences, The George Washington University, Lisner Hall, 2023 G Street, NW, Suite 340, Washington, DC 20052, USA; 4 Jerome Canady Research Institute for Advanced Biological and Technological Sciences, 6930 Carroll Avenue, 3rd floor Suite 300 Takoma Park, Maryland 20912, USA; 5 Neurological Surgery, The George Washington University, Foggy Bottom South Pavilion, 22nd Street, NW, 7th Floor, Washington, DC 20037, USA

## Abstract

To date, the significant anti-cancer capacity of cold atmospheric plasma (CAP) on dozens of cancer cell lines has been demonstrated *in vitro* and in mice models. Conventionally, CAP was directly applied to irradiate cancer cells or tumor tissue. Over past three years, the CAP irradiated media was also found to kill cancer cells as effectively as the direct CAP treatment. As a novel strategy, using the CAP stimulated (CAPs) media has become a promising anti-cancer tool. In this study, we demonstrated several principles to optimize the anti-cancer capacity of the CAPs media on glioblastoma cells and breast cancer cells. Specifically, using larger wells on a multi-well plate, smaller gaps between the plasma source and the media, and smaller media volume enabled us to obtain a stronger anti-cancer CAPs media composition without increasing the treatment time. Furthermore, cysteine was the main target of effective reactive species in the CAPs media. Glioblastoma cells were more resistant to the CAPs media than breast cancer cells. Glioblastoma cells consumed the effective reactive species faster than breast cancer cells did. In contrast to nitric oxide, hydrogen peroxide was more likely to be the effective reactive species.

During the past decade, cold atmospheric plasma (CAP), a near room temperature plasma mainly composed of reactive oxygen species (ROS) and reactive nitrogen species (RNS)^1^, has been extensively investigated for its promising application in anti-cancer therapy^2^,^3^. So far, CAP has shown a significant anti-cancer capacity over a wide range of cancer cell lines, including carcinomas^4^,^5^, melanomas^6^,^7^, neuroectodermal malignancies^8^,^9^, and hematopoietic malignancies^10^,^11^. In addition, the CAP also strongly resists tumor growth in mice^12^,^13^. Several general conclusions about the anti-cancer mechanism of CAP have been acknowledged. First, the rise of intracellular ROS always occurs in cancer cells upon CAP treatment^4^,^14^, which causes a noticeable damage on the antioxidant system^15^,^16^ and subsequently DNA double strands break (DSB) to a fatal degree^1^,^17^. Second, serious DNA damage and other effect of CAP on cancer cells result in the cell cycle arrest^18^, apoptosis or necrosis with a dose-dependent pattern^19^,^20^. Third, among diverse reactive species generated in CAP, H_2_O_2_ and NO are proposed to be key molecules to kill cancer cells^5^,^21^. Fourth, untransformed normal cells always show stronger resistance to CAP than cancer cells do^12^,^22^. Such killing preference on cancer cells is always accompanied with the distinct ROS levels and DSB among cancer cells and normal cells^23^,^24^.

Conventionally, the CAP is directly used to irradiate cancer cells or tissue. Over past three years, the CAP irradiated media was also found to kill cancer cells as effectively as the direct CAP treatment did^8^,^25^. In contrast to the direct CAP treatment, the CAPs media has advantages. The CAPs media can be stored in the refrigerator and maintain its anti-cancer capacity for at least 7 days^26^. Thus, the CAPs media might be a good fit for the condition where a CAP device is not available. Moreover the CAPs media can be injected into tissues and effectively prevent tumor growth^27^. These tissues may not be easily penetrated by the CAP jet, which only causes the cell death in the upper 3–5 cell layers of the CAP touched tissues^28^. To date, the anti-tumor capacity of the CAPs media has been researched less than the direct CAP treatment. Therefore, basic principles to guide its application remain elusive.

In this study, four factors have been found to be capable of optimizing the anti-cancer capacity of the CAPs media on glioblastoma cells (U87), breast cancer cells (MDA-MB-231 and MCF-7): the treatment time, the well size, the gap between plasma source and liquid, and the volume of media,. Glioblastoma is the most lethal form of brain cancer^29^. Due to its strong resistance to conventional therapy, the median survival time of patients is only 15 months^29^,^30^. CAP has shown promising anti-cancer capacity on glioblastoma cells *in vitro*^8^ and *in vivo*^13^. Breast cancer is the most common women malignancy in United States^31^. Estrogen receptor-negative MDA-MB-231 cells and estrogen receptor-positive MCF-7 cells are highly invasive and poorly invasive breast cancer cells, respectively^32^. The vulnerability of these three cell lines to the CAPs media was compared. In addition, we investigated which amino acids reacted most significantly with the effective reactive species by using the amino acids rich DMEM and the cell probes we invented. It was determined that, compared with NO, H_2_O_2_ was more likely to be main effective reactive species. Because the diffusion speed of H_2_O_2_ across the cellular membrane might directly affect the intracellular ROS level, the consumption speeds of effective reactive species and H_2_O_2_ by cancer cells were studied. Ultimately, the anti-cancer effect of H_2_O_2_ rich DMEM was investigated to explore whether H_2_O_2_ was the only effective reactive species. All detailed protocols were illustrated in Methods. This study built principles to guide the application of the CAPs media and provided insight to understanding the anti-cancer capacity of the CAPs media.

## Results

### CAP device and general research strategy

**Figure 1:**
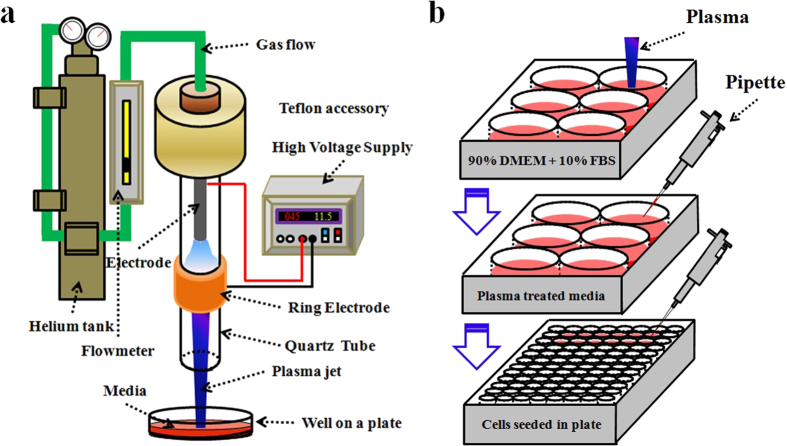
Schematic of the CAP device setup (**a**) and the general research strategy (**b**) to make the CAPs media.

The CAP device was a typical cold plasma jet generator using helium as the carrying gas, which has been used in the study about the CAP effect on the cell surface integrins expression^33^ and the response of glioblastoma cells upon the CAP treatment^9^,^34^. As shown in Fig. 1a, CAP was generated between central electrode and ring electrode and flowed out of the quartz tube via the helium gas. A flow meter controlled the helium flow at a rate of 4.7 L/min. The input voltage of DC power was 11.5 V. The output voltage was 3.16 kV. The power supply was about 5 W.

The general research strategy is illustrated in Fig. 1b. A plasma jet vertically irradiated the media in the wells in a 6-well plate. After treatment, the CAPs media was transferred to culture cancer cells, which had been seeded in a 96-well plate. Before this step, the initial media which had been used to culture cancer cells overnight was discarded. Then, cancer cells were cultured in the incubator under the standard conditions (a 37 °C, 5% (v/v) CO_2_ and humidified environment) for 72 hours. MTT (3-(4,5-dimethylthiazol-2-yl)-2,5-diphenyltetrazolium bromide), a colorimetric assay (Sigma-Aldrich) was harnessed to qualify the cell viability.

### The reactive species accumulate in the CAPs media with a dose-dependent and liquid surface-dependent pattern

Among dozens of species, NO in RNS and H_2_O_2_ in ROS are thought to play key roles in killing cancer cells^5^,^35^. In addition, hydroxyl free radicals (.OH) in CAP are also proposed to kill cancer cells^36^. To date, most of these conclusions are based on the research for the direct CAP treatment on cancer cells. The understanding on the RNS and ROS accumulation in the CAPs media is far from clear. In this study, we first studied the generation of NO and H_2_O_2_ in the CAPs complete media (90% DMEM + 10% FBS) via Griess Reagent System (Promega) and Fluorimetric Hydrogen Peroxide Assay Kit (Sigma-Aldrich), respectively.

Next, we harnessed methylene blue (Fisher) to qualify the generation of .OH. In contrast to terephthalic acid, which has also been used to qualify the .OH generation after plasma treatment^36^, methylene blue (MB) is a cheaper and more convenient probe. MB strongly reacts with .OH in the aqueous solution and changes its color from blue into colorless, which can be detected by spectrophotometer at 664 nm^37^,^38^. The atmospheric plasma jet is a proven MB decomposition tool^39^. We proved that MB was just sensitive to the species with a short half-life (Fig. S1). Because MB is strongly absorbed by the proteins in the complete media, we investigated the generation of .OH in the CAPs deionized water in this study.

**Figure 2:**
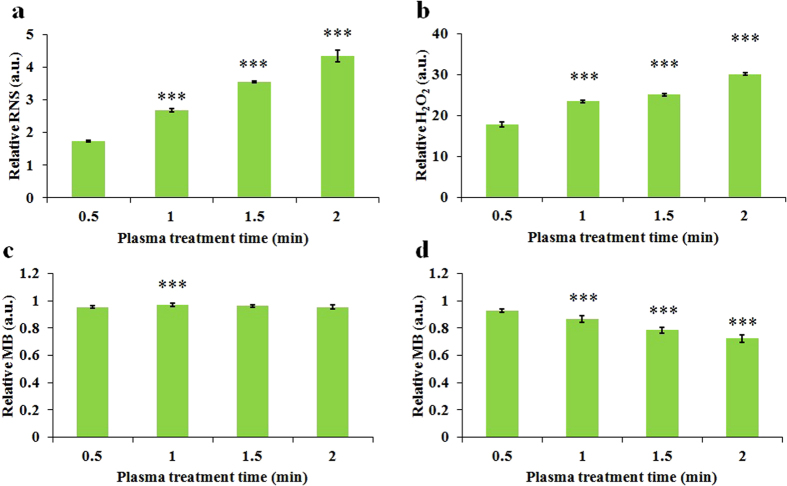
The dose-dependent ROS/RNS accumulation in the CAPs solution. (**a**) Relative RNS concentration in 1 mL CAPs complete media. (**b**) Relative H_2_O_2_ concentration in 1 mL CAPs complete media. (**c**) Relative MB concentration in 1 mL CAPs MB solution. (**d**) Relative MB concentration in 2 mL CAPs MB solution. Results are presented as the mean ± s.d. of three repeated experiments performed in triplicate. Student’s t-test was performed, and the significance compared with the first bar is indicated as *p < 0.05, **p < 0.01, ***p < 0.005.

Reactive species accumulate in the CAPs media with different patterns. To better illustrate these patterns, all data in Fig. 2 have been normalized to be the relative values via dividing the data from experimental group by the data from corresponding control group. RNS (Fig. 2a) and H_2_O_2_ (Fig. 2b) in the CAPs media both increase as the treatment time increases. Because MB will be consumed after the reaction with .OH, corresponding relative absorbance of the CAPs MB solution should be less than 1 (Fig. S1). However, as shown in Fig. 2c, noticeable .OH generation was not observed even the treatment time was extended to 2 min. We performed the same experiment in the MB solution with a larger volume (2 mL) and found that the generation of .OH was proportional to the plasma treatment time again (Fig. 2d). This volume-dependent pattern may be due to the interaction between plasma jet and solution. During the CAP treatment, the liquid below the plasma jet would be extruded, pushing the solution to the perimeter of well (Fig. S2a). When the volume of liquid was just 1 mL, the plasma jet could not touch liquid due to the exposure of the bottom of well. When the volume of liquid was up to 2 mL, the extrusion of liquid would be weaker and enabled the plasma jet to touch the liquid layer. However, even when the plasma jet does not touch the liquid, the reactive species are still able to dissolve into the liquid. Yonemori *et al.* observed that when an atmospheric-pressure plasma jet touched a glass surface, it flowed radically over the glass surface and formed a large area containing reactive species on the glass surface^40^. Thus, the reactive species in the plasma jet should affect an area of liquid that is significantly larger than the diameter of the jet. The half-life of .OH is only a few microseconds^41^, however, which eliminates the possibility that .OH diffuses over the liquid surface (Fig. S2a). In contrast, H_2_O_2_ and NO with much longer half-life may enter the media by the diffusion over the whole surface of liquid. We denote that H_2_O_2_/NO area and .OH area to represent the area mainly affected by H_2_O_2_/NO and .OH on the liquid surface covered by plasma flow, respectively (Fig. S2a). Together, when the volume of media is relative small, .OH will not be a main factor to directly affect the anti-tumor capacity of the CAPs media. .OH may react with .OH to form H_2_O_2_ and affects the anti-tumor capacity indirectly.

**Figure 3:**
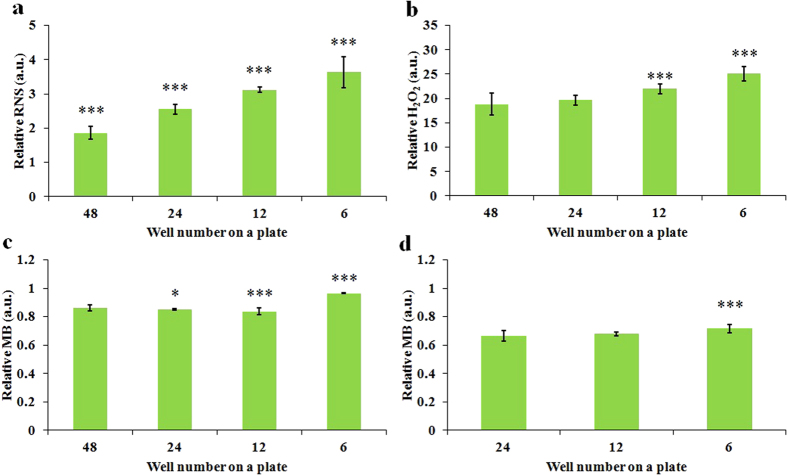
The well size-dependent ROS/RNS accumulation in the CAPs solution. (**a**) Relative RNS concentration in 1 mL of CAPs complete media. (**b**) Relative H_2_O_2_ concentration in 1 mL of CAPs complete media. (**c**) Relative MB concentration in 1 mL of CAPs MB solution. (**d**) Relative MB concentration in 2 mL CAPs of MB solution. Results are presented as the mean ± s.d. of three repeated experiments performed in triplicate. Student’s t-test was performed, and the significance compared with the first bar is indicated as *p < 0.05, **p < 0.01, ***p < 0.005.

If H_2_O_2_/NO area and .OH area do exist as we depicted in Fig. S2, it is reasonable to deduce that the surface of media irradiated by the plasma may affect the accumulation of H_2_O_2_/NO but not .OH in the CAPs media. We proved this deduction by measuring the production of H_2_O_2_/NO and .OH in the CAPs media and CAPs MB solution in distinct multi-well plates, respectively. The well diameters on 48-well, 24-well, 12-well, and 6-well plate were 10.2 mm, 15.4 mm, 21.4 mm, and 35.0 mm, respectively. We found that the H_2_O_2_/NO concentration in the CAPs media varied significantly with the well size on the plate. The larger diameter of well, the more H_2_O_2_/NO accumulated in the CAPs media (Fig. 3a,b). By contrast, the .OH generation in the CAPs MB solution doesn’t significantly vary with the well size (Fig. 3c), even when the volume of MB solution is up to 2 mL (Fig. 3d). The .OH generation in the CAPs MB solution from a 6-well plate is noticeably lower than that from other plates (Fig. 3c).

A schematic illustration in Fig. S2b depicts the underlying mechanism of the well size-effect on the reactive species accumulation. A smaller well creates a smaller H_2_O_2_/NO area on the media surface. More H_2_O_2_/NO will diffuse into the CAPs media when the well size becomes larger. Additionally, the thickness of media in the well increases as the well size decreases. The calculated liquid thickness of 1 mL media in 48-well, 24-well, 12-well, 6-well plate are 12.2 mm, 5.4 mm, 2.8 mm, and 1.0 mm, respectively (Fig. S2b). Thus, the extrusion of media due to the plasma jet pressure will be weakened as the diameter of well decreases. When the volume of MB solution is just 1 mL, compared with the MB solution in 6-well plate, the MB solution in other multi-well plates is more likely to contact the .OH in the CAP. Thus, more MB is consumed in the CAPs MB solution from 48-well, 24-well, and 12-well plate than that from 6-well plate (Fig. 3c). Even when the volume of MB solution is 2 mL, .OH is only able to affect a small area on the media directly touched by the CAP jet, so the .OH generation in the CAPs MB solution changes little when the well size is noticeably altered. In short, the distinct half-life among H_2_O_2_/NO and .OH underlies the different well size-effect of reactive species observed in this study.

### The anti-tumor capacity of the CAPs media is dose-dependent and well size-dependent

**Figure 4:**
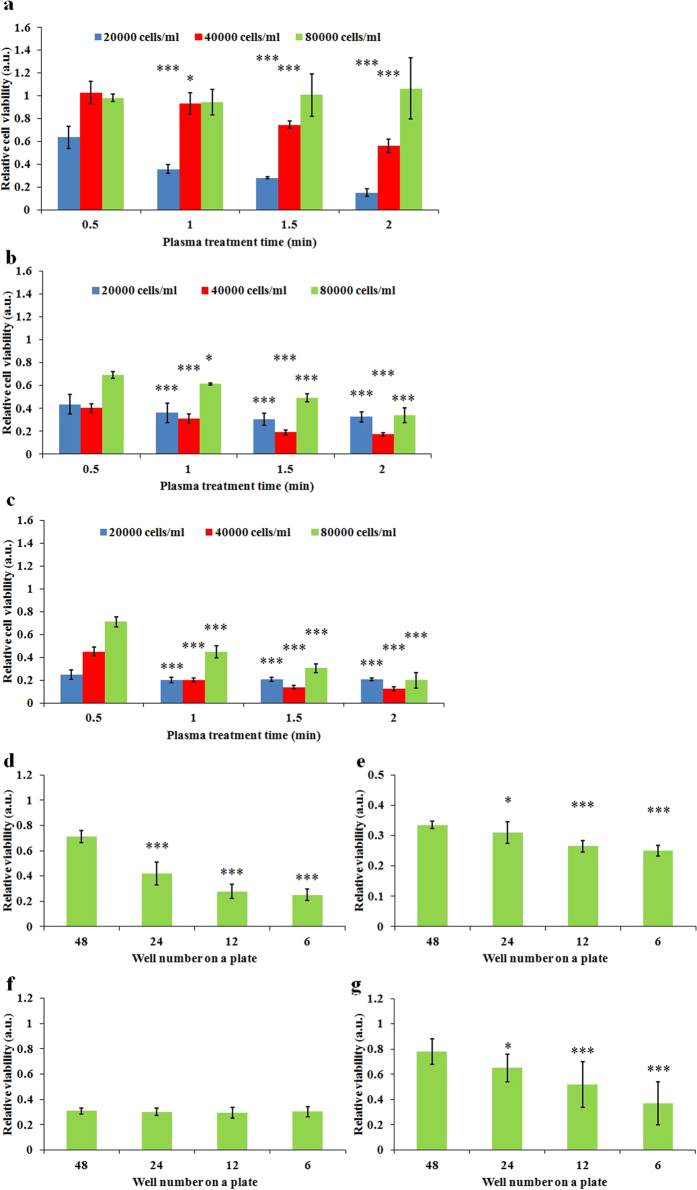
The dose-dependent and well size-dependent anti-cancer capacity of the CAPs solution. The relative viability of U87 cells (**a**), MDA-MB-231 cells (**b**), and MCF-7 cells (**c**) cultured in 1 mL of CAPs media with different treatment time. (**d**) The relative viability of U87 cells (2 × 10^4^ cells/ml) (**d**), MDA-MB-231 cells (2 × 10^4^ cells/ml) (**e**), MCF-7 cells (2 × 10^4^ cells/ml) (**f**), and MCF-7 cells (4 × 10^4^ cells/ml) (**g**) cultured in 1 mL of CAPs media from different multi-well plates. The treatment time for (**d–g**) were 1 min. Results are presented as the mean ± s.d. of three repeated experiments performed in sextuplicate. Student’s t-test was performed, and the significance compared with the first bar is indicated as *p < 0.05, **p < 0.01, ***p < 0.005.

We further investigated the anti-tumor capacity of CAPs media on glioblastoma cells (U87), breast cancer cells (MDA-MB-231 and MCF-7) with distinct cell confluences. It was found that the anti-tumor capacity of the CAPs media increases as the treatment time (dose) increases and decreases as the cell seeding confluence decreases (Fig. 4a–c). Thus, it is the dose of CAP treatment exerting on a unit cell, rather than the whole CAP treatment dose, that determines the fate of cancer cells. In addition, MDA-MB-231 cells and MCF-7 cells are more vulnerable to the CAPs media than U87 cells. The response of MDA-MB-231 cells and MCF-7 cells to the CAPs media is similar, though MCF-7 cells are a little easier to be killed.

We further investigated effect of well size on the anti-tumor capacity of the CAPs media on the same three cancer cell lines. We found that the anti-tumor capacity of the CAPs media decreased as the size of the wells decreased (Fig. 4d–g). For U87 cells, the residual viability of cells cultured in the CAPs media from a 6-well plate is about 1/3 of the viability of cells cultured in the CAPs media from a 48-well plate (Fig. 4d). In other words, at least 2/3 of the anti-cancer ability of the CAPs media is wasted in the 48-well plate. A similar trend is also observed on MDA-MB-231 cells (Fig. 4e). However, the well size-effect on MCF-7 cells only appears when the seeding confluence is as high as 4 × 10^4^ cells/ml (Fig. 4g), which may be due to the fact that even the CAPs media from a 48-well plate is adequate to kill almost all MCF-7 cells with a seeding confluence as low as 2 × 10^4^ cells/ml (Fig. 4f). Accordingly, the 96-well plate should waste more reactive species. By surveying all publications about the application of plasma jet on cancer treatment, it was determined that only 25% publications used 6-well plate or dish with same size in their CAP treatment (Fig. S3). Thus, most investigations wasted significant amount of plasma, gas and media over the past decade. The dose-dependent and the well size-dependent anti-cancer features of the CAPs media is consisted with the dose-dependent and liquid surface-dependent reactive species accumulation patterns in the CAPs media.

### The anti-tumor capacity of the CAPs media varies with the gap between plasma source and media

**Figure 5:**
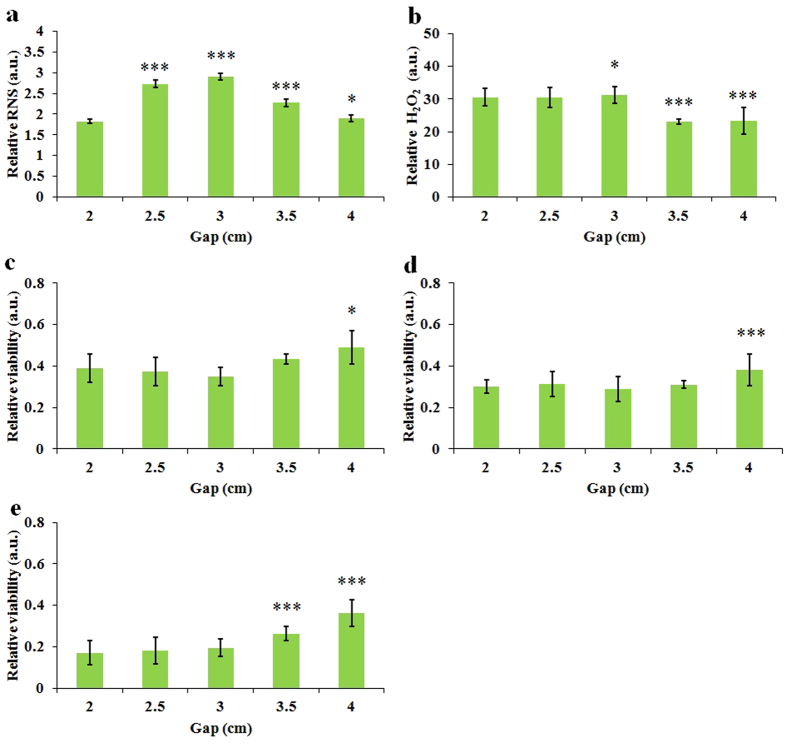
The ROS/RNS accumulation in the CAPs media and the anti-cancer capacity of CAPs media are gap-dependent. (**a**) Relative RNS concentration in 1 mL CAPs complete media. (**b**) Relative H_2_O_2_ concentration in 1 mL of CAPs complete media. (**c**) Relative viability of U87 cells (**c**), MDA-MB-231 cells (**d**), and MCF-7 cells (**e**) cultured in 1 mL of CAPs complete media. The treatment time for all figures was 1 min. Results are presented as the mean ± s.d. of three repeated experiments performed in triplicate (**a,b**) or in sextuplicate (**c,d,e**). Student’s t-test was performed, and the significance compared with the first bar is indicated as *p < 0.05, **p < 0.01, ***p < 0.005.

For the media in the well with identical volume, when the well size decreases, the height of the media in the well increases. Thus, the well size-dependent effect may be due to the altered size of the gap between the surface of the media and the plasma source. We investigated the generation of NO and H_2_O_2_ in CAPs media by altering the gap between the bottom of plate and the nozzle of quartz tube. We found that the NO and H_2_O_2_ in the CAPs media shown a distinct response to a change in gap. As the gap increases from 2 cm to 4 cm, the concentration of NO in the CAPs media shows a parabolic response. It reaches a peak at the gap of 3 cm and then decreases as the gap is increased to 4 cm (Fig. 5a). The distribution of NO along the axial direction of the atmospheric pressure plasma jet has been measured by laser induced fluorescence and showed similar trend as we observed^42^. In contrast, the concentration of H_2_O_2_ shows a stepwise change upon the gap increase. The concentration of H_2_O_2_ in the CAPs media remains fairly constant over the gap of 2 cm to 3 cm. When the gap increased from 3 cm to 4 cm, the concentration of H_2_O_2_ in the CAPs media decreased about 26% (Fig. 5b). Next, we investigated the viability change of three cancer cell lines upon the gap change. As shown in Fig. 5c–e, the anti-tumor capacity of the CAPs media dose not noticeably change until the gap increases to 3.5 cm or 4 cm. The gap-effect on the anti-cancer capacity is consistent with the gap-effect on the generation of H_2_O_2_ but significantly differs from the gap-effect on the generation of NO. It indicates that H_2_O_2_ rather than NO may dominate the death of cancer cells. Ultimately, Fig. 5 proves that the well size-effect on the CAPs media is not due the gap-effect. Because the smaller gap only tends to generate more reactive species, the actual well size-effect should be stronger than that shown in Fig. 4.

### The anti-cancer capacity is volume-dependent

**Figure 6:**
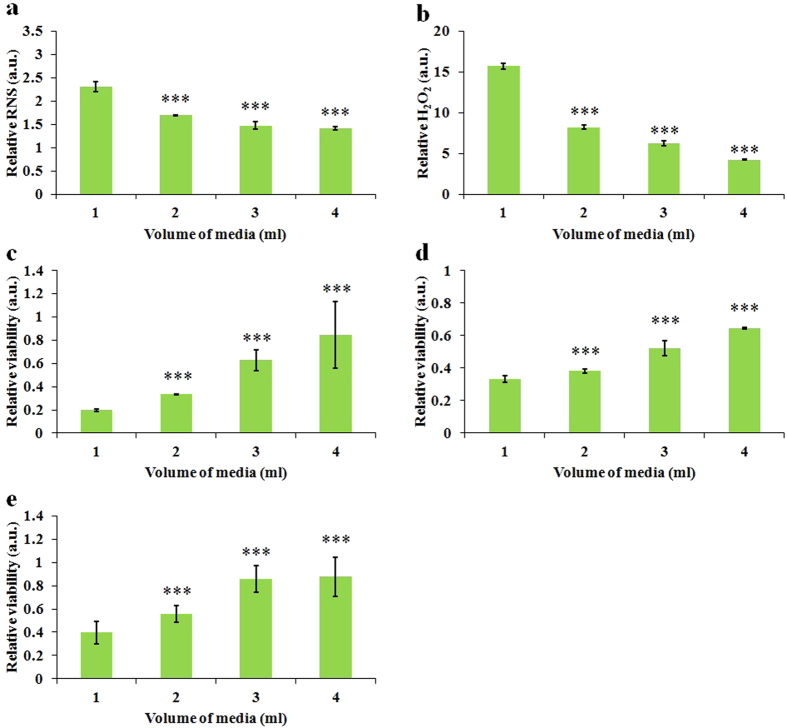
The ROS/RNS accumulation in the CAPs media and the anti-cancer capacity of CAPs media are volume-dependent. (**a**) Relative RNS concentration in the CAPs complete media. (**b**) Relative H_2_O_2_ concentration in the CAPs complete media. (**c**) Relative viability of U87 cells (**c**), MDA-MB-231 cells (**d**), and MCF-7 cells (**e**) cultured in the CAPs complete media. The treatment time for all figures was 1 min. Results are presented as the mean ± s.d. of three repeated experiments performed in triplicate (**a,b**) or in sextuplicate (**c,d,e**). Student’s t-test was performed, and the significance compared with the first bar is indicated as *p < 0.05, **p < 0.01, ***p < 0.005.

As early as 2007, it was found that melanoma cells could be protected in FE-DBD treatment by varying depth of cell growth media^3^. However, the mechanism underlying the protecting role of media remains obscure. As we have demonstrated, the strong anti-cancer capacity of the CAPs media always accompanies a high concentration of species in the media. Because the increase of media depth means the increase of media volume in the same container, the protecting role of media may just due to the dilution of reactive species in the media. We investigated the volume-effect on the anti-cancer capacity of the CAPs media. It was found that both the concentrations of H_2_O_2_ (Fig. 6a) and NO (Fig. 6b) decreased as the volume of media increased. Furthermore, the killing capacities of the CAPs media on three cancer cell lines significantly decreased as the volume of media increased (Fig. 6c–e). In short, the protecting role of media is due to the dilution-effect of the reactive species. According to this principle, the optimized anti-cancer capacity of CAP treatment, direct or indirect, can be achieved when cells are surrounded by a few media.

### Cysteine and tryptophan are the main targets of effective species in the CAPs media

Here, we denote effective species to represent the effective reactive species which kill cancer cells. Understanding the chemical essence of the reaction between the effective species and the intracellular molecules is a prerequisite to understanding the mechanism underlying the anti-cancer capacity of CAP treatment. However, to study the intracellular reaction between RNS/ROS with the thousands of intracellular molecules directly is too challenging to be performed. In this study, we focused on revealing which amino acids significantly reacted with the effective species. The interaction between CAP and the amino acid solution have been studied via mass spectra. It was found that the sulfur-containing and aromatic amino acids in the aqueous solution were preferentially consumed in the CAP treatment^43^. Methionine, cysteine, tryptophan, phenylalanine, and tyrosine were the five most consumed reactive amino acids upon the CAP treatment^43^. However, some amino acids may be consumed by the species which do not kill cancer cells. The revealed reaction types between amino acids and plasma have not answered the question of which amino acids tend to react with the effective species.

We harnessed a novel strategy to reveal the reaction strength among effective species and specific amino acids. We used cancer cells as a cell probe to investigate whether the effective species in the CAPs media would be consumed by particular amino acids via comparing the anti-cancer capacity of a specific 2.4 mM CAPs amino acids rich DMEM with the corresponding untreated amino acids rich DMEM. Each amino acids rich DMEM was prepared by dissolving specific amino acids powers in DMEM.

**Figure 7:**
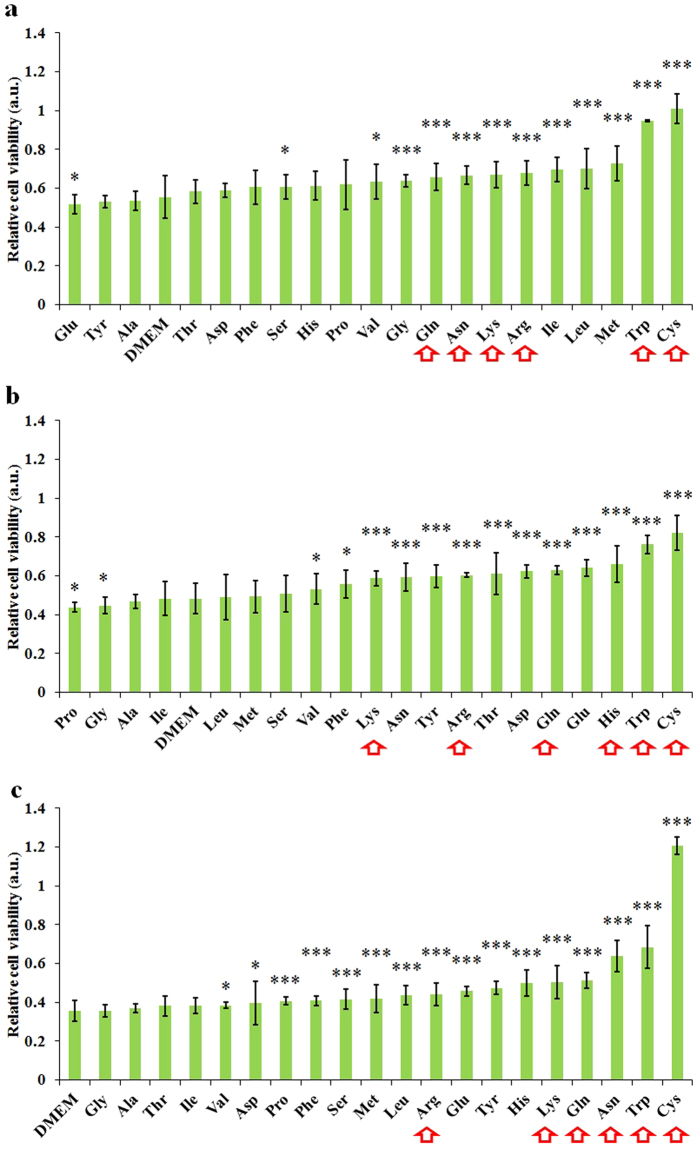
Cysteine and tryptophan are the most reactive amino acids towards the effective species in the CAPs DMEM. Relative cell viability of U87 cells (**a**), MDA-MB-231 cells (**b**), and MCF-7 cells (**c**) cultured in the CAPs amino acids rich DMEM (2.4 mM). The treatment time for all figures was 1 min. Results are presented as the mean ± s.d. of three repeated experiments performed in sextuplicate. Student’s t-test was performed, and the significance compared with the bar of DMEM is indicated as *p < 0.05, **p < 0.01, ***p < 0.005. Red arrows mark the amino acids which are significantly weaken the anti-cancer capacity of CAPs media for three cell lines.

Among 20 amino acids, cysteine and tryptophan showed the strongest reactivity towards the effective species in CAPs media. In contrast to DMEM, the cysteine rich DMEM consumes most effective species and almost completely eliminates the anti-tumor capacity of the CAPs DMEM on U87 cells (Fig. 7a), MDA-MB-231 cells (Fig. 7b), and MCF-7 cells (Fig. 7c). Tryptophan has a similar but weaker capacity to consume effective species. A control group was also analyzed for each amino acid tested, because if a specific amino acid rich DMEM is toxic to cancer cells, the corresponding control group has a very low cell viability, which makes the relative cell viability in Fig. 7 appears very large. As a result, we may get a delusion that a toxic amino acid consumes most effective species. In this study, for U87 cells and MCF-7 cells, tryptophan shows a strong resistance to growth of cancer cells (Fig. S4a,c). The mechanism underlying this toxicity is unclear. We also found that the tryptophan-rich DMEM did not resist the growth of MDA-MB-231 cells (Fig. S4b). Thus, the weak anti-tumor capacity of the CAPs tryptophan rich DMEM on MDA-MB-231 cells conclusively demonstrates that tryptophan is the second most sensitive amino acid to the effective species. In addition, the CAP treated DMEM tends to cause the most cell death among most CAPs amino acid rich DMEM. Thus, the effective species in CAP reacts with wide range of amino acids. Other than cysteine and tryptophan, arginine, lysine, asparagine, and glutamine also react significantly with the effective species in the CAPs media. Thus, they should also be the hot targets of effective species.

**Figure 8:**
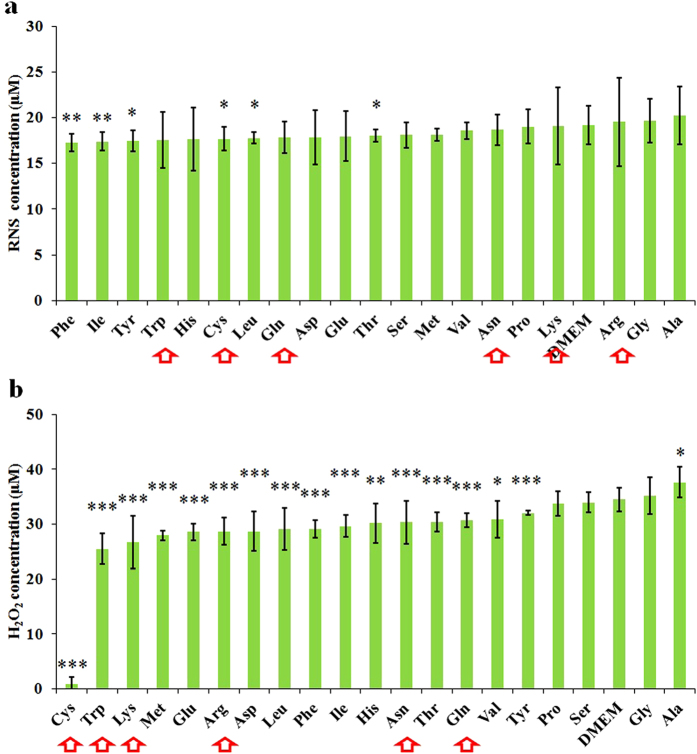
In contrast with NO, H_2_O_2_ is more reactive with amino acids. The concentration of RNS (**a**) and H_2_O_2_ (**b**) in the CAPs amino acids rich DMEM (2.4 mM). (**a**) is presented as the mean ± s.d. of three repeated experiments performed in sextuplicate. (**b**) is presented as the mean ± s.d. of two repeated experiments performed in sextuplicate. Student’s t-test was performed, and the significance compared with the bar of DMEM is indicated as *p < 0.05, **p < 0.01, ***p < 0.005. Red arrows mark the amino acids which are significantly weaken the anti-cancer capacity of CAPs media for three cell lines.

Furthermore, we studied the generation of H_2_O_2_ and NO in the CAPs amino acid rich DMEM, which will reveal the species reacting strongly with specific amino acids. In contrast to NO, H_2_O_2_ is more likely to be the effective species, because no significant difference of NO generation exists between different CAPs amino acids rich DMEM (Fig. 8a), while H_2_O_2_ in the CAP simulated amino acid DMEM varied specifically with the amino acids in DMEM. Most of the hot targeted amino acids of effective species are also capable of consuming H_2_O_2_ significantly in the CAPs DMEM (Fig. 8b). There are 15 amino acids that consume the H_2_O_2_ generated in the CAPs DMEM, which is consistent with the conclusion made above that many amino acids react with the effective species in the CAP treated DMEM. Among them, cysteine rich DMEM consumes almost all H_2_O_2_. Despite tryptophan is the second most sensitive amino acids to H_2_O_2_, it just consumes about 23% of H_2_O_2_ as cysteine does.

### Cancer cells consume the reactive species with a cell line-dependent pattern

**Figure 9:**
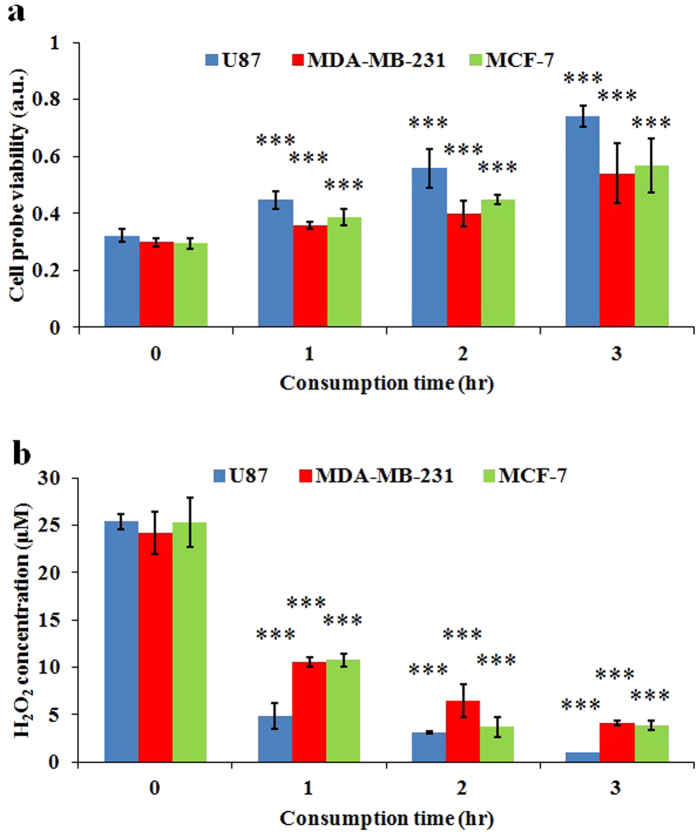
The consumption speed of effective species in the CAPs media by cancer cells. (**a**) Cell probe (MDA-MB-231 cells) was used to discriminate how fast the effective species in the CAPs media consumed by three cancer cell lines. The cell probe viability in this figure represents the ratio of the viability of cell probe (MDA-MB-231 cells) cultured in the residual CAPs media which has been used to culture U87 cells, MDA-MB-231 cells, and MCF-7 cells for a period of time to the viability of cell probe (MDA-MB-231 cells) cultured in the complete media without CAP treatment. (**b**) The H_2_O_2_ concentration in the residual CAPs media which has been used to culture U87 cell, MDA-MB-231 cells, and MCF-7 cells for a period of time. Results are presented as the mean ± s.d. of three repeated experiments performed in sextuplicate (**a**) or triplicate (**b**). Student’s t-test was performed, and the significance compared with the first bar (consumption time of 0 hr) is indicated as *p < 0.05, **p < 0.01, ***p < 0.005.

Due to the obscure essence of the effective species in the CAP, the consumption speed of the effective species by cancer cells has not been reported. Briefly, in this study, we harnessed one cancer cell line (MDA-MB-231) as the cell probe to investigate how much the effective species were left in the CAPs media which had been used to culture three cancer cell lines for a period of time (Fig. S5). We denoted such CAPs media and such time as residual media and consumption time, respectively. The viability of cell probes (MDA-MB-231) was inversely proportional to the concentration of residual effective species in the residual media. The detailed protocols were illustrated in Fig. S5. It has been found that the effective species in the residual media are gradually consumed by all three cell lines, which causes the viability of cell probe (MDA-MB-231 cells) cultured in residual media to increase as the consumption time increases (Fig. 9a). In addition, the cell probe (MDA-MB-231 cells) cultured in the residual media which has been used to culture U87 cells obtains higher cell viability than the cell probe cultured in the residual media which has been used to culture MDA-MB-231 cells and MCF-7 cells (Fig. 9a). In other words, U87 cells consume the effective species significantly faster than MDA-MB-231 cells and MCF-7 cells. For U87 cells, the residual CAPs media almost completely lose its anti-cancer capacity 3 hours after treatment. In contrast, for MDA-MB-231 cells and MCF-7 cells, the CAPs media still maintain significant anti-cancer capacity 3 hours after treatment. MDA-MB-231 cells and MCF-7 cells consumes the effective species similarly.

Because H_2_O_2_ was regarded as the main effective species in the CAPs media, we further investigated the decay speed of H_2_O_2_ in the residual media by measuring the evolution of H_2_O_2_ in the CAPs media which had been used to culture three cell lines. We found that the H_2_O_2_ in the residual media which had been used to culture U87 cells decayed noticeably faster than the cells in the residual media which had been used to culture MDA-MB-231 cells and MCF-7 cells (Fig. 9b). Thus, U87 cells are capable of consuming H_2_O_2_ faster than MDA-MB-231 cells and MCF-7 cells.

### H_2_O_2_ alone does not equal to the CAPs media

**Figure 10:**
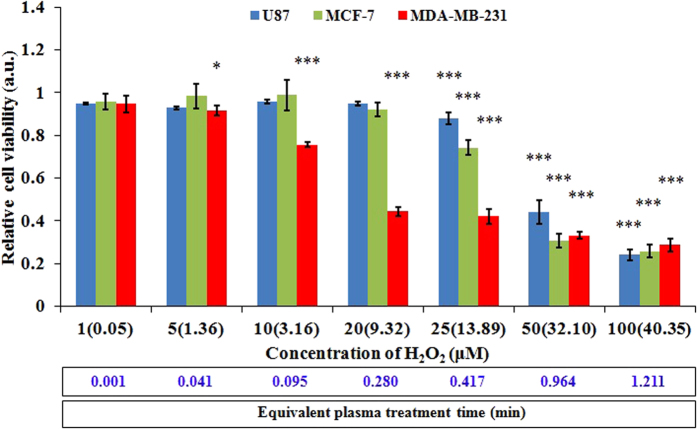
The anti-cancer capacity of the H_2_O_2_ rich media on U87 cells, MDA-MB-231 cells, and MCF-7 cells. The real concentration measured by Fluorimetric Hydrogen Peroxide Assay Kit (Sigma-Aldrich) is shown in the parenthesis following the nominal concentration based on the calculation in preparation. The equivalent CAP treatment time (eqT) is shown in the bottom of figure. eqT was calculated based on the formula that eqT = (measured H_2_O_2_ concentration in the H_2_O_2_ rich media, mC) × (conversion coefficient, cC). Here, cC is 0.03. For example, eqT of 1.211 = mC of 40.35 times cC of 0.03. To obtain cC, we first measured the H_2_O_2_ concentration in the media which has been treated by CAP for 0.5, 1, 1.5, and 2 min. Then, the linear fitting between the measured H_2_O_2_ concentration and the treatment time helped us to get cC. Results are presented as the mean ± s.d. of three repeated experiments performed in sextuplicate. Student’s t-test was performed, and the significance compared with the first bar (nominal concentration of 1 μM) is indicated as *p < 0.05, **p < 0.01, ***p < 0.005.

To investigate whether H_2_O_2_ was the sole factor in causing the death of cancer cells, we studied the response of three cancer cell lines to the H_2_O_2_ rich media. The H_2_O_2_ rich media was prepared by adding 30 wt % H_2_O_2_ solution (Sigma-Aldrich) into the complete media. Because H_2_O_2_ reacted with proteins in the complete media, the real concentration measured by Fluorimetric Hydrogen Peroxide Assay Kit (Sigma-Aldrich) was much less than the nominal concentration based on calculation in preparation. As shown in Fig. 10, the growth of three cell lines presents a similar concentration (dose)-dependent response to the H_2_O_2_ rich media. The growth of three cell lines will not be drastically suppressed until the concentration of H_2_O_2_ is adequately large. It is not surprising, because high dose of H_2_O_2_ produces cell death^44^. However, the responses of three cancer cells to H_2_O_2_ are quite different. Generally, U87 cells and MCF-7 cells show stronger resistance to H_2_O_2_ than MDA-MB-231 cells. U87 cells and MCF-7 cells share a similar response to H_2_O_2_ except when the nominal concentration is between 20 to 50 μM. Clearly, the selective anti-tumor capacity of H_2_O_2_ on three cells lines are distinct from that of the CAPs media on these cell lines (Fig. 4 and Fig. S6). U87 cells are more resistant to the CAPs media than both MCF-7 cells and MDA-MB-231 cells. MDA-MB-231 cells are a slightly more resistant to the CAPs media than MCF-7 cells. These differences demonstrate that H_2_O_2_ is not the only reactive specie to cause the death of cancer cells. Some other species also play necessary role. Cysteine may react with not only H_2_O_2_ but also other effective species in the CAPs media.

## Discussion

So far, the anti-tumor capacity of CAP treatment has been regulated by controlling the treatment time^3^, the gas sources composition^34^,^45^, the gas flow rate^34^, and the supply voltages^46^. Gold nanoparticles^6^,^9^ and some small molecules such as osmolytes^47^ and 2-deoxy-d-glucose^48^ were also used to obtain a synergistic anti-cancer effect. In this study, we demonstrated several methods to obtain stronger anti-cancer capacity of the CAPs media. Specifically, the CAP treatment should be performed in a container with a large diameter. In addition, the gap between the plasma source and the media should be adequately small. To obtain high reactive species concentration, the volume of media should be relative small. Because these principles are fit for all cell lines involved in this study, they may be universal for other cancer cells.

Because 20 amino acids are the building blocks of all proteins, cysteine, the most reactive amino acid determined by cell probes, should also be the main targeted amino acid residue on the intracellular proteins or other small molecules. Intracellular ROS levels and redox balance are tightly regulated by multiple antioxidant defense systems, including small antioxidant molecules such as glutathione, NAD(P)H and ROS-scavenging enzymes such as catalase, superoxide dismutase, glutathione peroxidase, glutathione reductase, thioredoxin, thioredoxin reductase, and peroxiredoxin^49^. Redox status of thiol group on the cysteine residue directly determines the function of glutathione peroxidase^50^, thioredoxin^51^, thioredoxin reductase^52^,^53^, and peroxiredoxin^54^. When thiold group on the cysteine residue was oxidized, corresponding normal function of some anti-oxidant enzymes or small molecules will be completely lost. Here, we take glutathione, the most abundant small molecular thiol inside mammalian cells^55^, as an example. Reduced form of glutathione (GH) is a tripeptide with a cysteine residue in the middle. When GH is oxidized, the cysteine residue will form a disulfide bond with the cysteine residue on another GH. Such product is denoted as GSSG. GSSG loses the ability to be a anti-oxidant molecule^55^. Actually, the weakend antioxidant system has been observed in the plasma treated cancer cells^15^,^56^. The weakened intracellular anti-oxidant system facilitates the attack of extracellular ROS on cells, and ultimately results in serious ROS rise^4^,^24^ and oxidative damage including DSB^1^,^17^ and carbonyl content formation^15^.

Like other ROS, the toxicity of H_2_O_2_ to cells are dose-dependent. Only the high dose of H_2_O_2_ can produce cell death^44^. The toxicity of H_2_O_2_ is exaggerated by the Fenton reaction between H_2_O_2_ and Fe^2+^ in cells, which generates high reactive .OH and causes damage to DNA and other important intracellular molecules^57^. H_2_O_2_ has been proposed as a key species to cause the death of cancer cells after CAP treatment 21,26,35. However, the biochemical analysis on the cancer cells following the treatment of CAP and H_2_O_2_ demonstrates the distinct phosphorylation levels on c-Jun amino-terminal kinases and p38 protein kinases^35^. Our study revealed that the H_2_O_2_ rich media could not generate the same selective killing on cancer cell lines as well as the CAPs media did. Thus, the CAP treatment can not equate to the H_2_O_2_ treatment.

In contrast to H_2_O_2_, NO was not likely to be the main effective species. However, the real role of NO in killing cancer cells has not been deeply explored in this study. Theoritically, NO may result in the general observed ROS increase and the death of cancer cells after CAP treatment. NO is able to increase intracellular ROS levels by blocking the electron transport chain in mitochondria^58^ and inactivating the glutathione peroxidase^59^. NO reacts with superoxide to generate peroxynitrite, which will not only attack DNA but also weaken the antioxidant system via the inactivation of manganese-superoxide dismutase^60^,^61^, glutathione peroxidase^62^, glutathione reductase^63^,^64^, catalase^65^, and peroxiredoxin^66^. In addition, other effective species which have not been studied may also contribute to the death of cancer cells. Thus, the number of species that codetermine the anti-cancer capacity of CAPs media is still unknown.

Recently, two trends regarding the response of cancer cells to the CAP treatment have been found. First, the anti-tumor capacity of CAP on cancer cells is proportional to the growth speed of cancer cell lines^67^. Second, the cancer cells carrying mutated p53 genes are more vulnerable to the CAP treatment than the cancer cells carrying wild p53 genes^68^. The second trend might explain the first trend to some extent. Cancer cell lines carrying the mutated p53 gene tend to obtain malignant^69^ or metastasis phenotypes^70^,^71^ and overcome growth arrest and senescence^72^. The cancer cells in metastasis stage own prosperous metabolism and high ROS level^73^,^74^. Thus, the fast growing cancer cells without a normal, functional p53 gene are more vulnerable to the CAP treatment. In this study, however, we found a new trend that the cancer cells that could absorb or eliminate the effective species in the surrounding environment faster would be more resistant to the CAPs media. Despite the fact that the intrinsic relationship between these trends was obscure, we demonstrated that the absorption capacity on the effective species and H_2_O_2_ by cancer cells significantly varied with the cell lines.

The absorption of reactive species, mainly H_2_O_2_ in this study, directly relates to the diffusion speed of reactive species across the cellular membrane. H_2_O_2_ has been regarded as a molecule that is able to freely cross the phospholipid membrane^75^. Recent investigations revealed that diffusion of H_2_O_2_ across phospholipid membrane was limited by the membrane composition^75^. Due to the highly similarity among H_2_O_2_ and water, aquaporins (AQPs), a membrane protein family facilitating the transport of water across the cellular membrane, also plays an important role in facilitating the passive diffusion of H_2_O_2_^76^. Not all AQPs are able to transport H_2_O_2_. So far, only the transport of H_2_O_2_ by AQP1 and AQP8 has been investigated. AQP1 with a relative smaller pore diameter in the selectivity filter region (2.7 Å) cannot transport H_2_O_2_^75^,^77^. By contrast, due to the larger pore diameter (3.2 Å)^56^, AQP8 is able to transport H_2_O_2_ across the cellular membrane^78^. AQPs are expressed to varying degrees in different types of human tumors. For example, AQP1, 4, and 5 highly express in breast cancer cell lines^79^,^80^. AQP1, 4, 8, and 9 highly express in glioblastoma cell lines^81^,^82^,^83^. The distinct expression pattern of AQP8 in glioblastoma cells and breast cancer cells can explain why H_2_O_2_ is consumed faster by U87 cells than MCF-7 and MDA-MB-231. Nonetheless, if H_2_O_2_ is the main effective species to kill cancer cells, the intracellular H_2_O_2_ level should be at least codetermined by the diffusion speed of H_2_O_2_ across the cell membrane and the intracellular H_2_O_2_ scavenging system^75^. Thus, we may not be able to predict the vulnerability of cancer cells to the CAPs media only based on the distinct consumption speeds of cancer cells. More research focusing the intracellular H_2_O_2_ scavenging capacity in distinct cancer cells should be carried out in the future.

## Conclusions

In summary, we demonstrated several principles to optimize the anti-tumor capacity of the CAPs media on glioblastoma cells and breast cancer cells. Specifically, a larger well, a closer gap between plasma source and media, and a smaller volume of media produce a stronger anti-cancer CAPs media. Breast cancer cells are more vulnerable to the CAPs media than glioblastoma cells. In addition, compared with NO, H_2_O_2_ in the CAPs media is likely to be the main effective species to kill cancer cells. The effective species in the CAPs media mainly react with cysteine, which explains the rise of intracellular ROS in the CAP treated cancer cells. H_2_O_2_ and the CAPs media cause distinct selective killing patterns in cancer cells, indicating that other reactive species may also affect the death of cancer cells. Glioblastoma cells are able to consume effective species and H_2_O_2_ in the CAPs media significantly faster than breast cancer cells, which may relate to the distinct expressions of cell membrane proteins on cancer cells.

## Methods

### Cell cultures

Human U87 cells were provided by Dr. Murad’s Lab at the George Washington University. Human MDA-MB-231 cells and MCF-7 cells were provided by Dr. Zhang’s Lab at the George Washington University. All cancer cell lines were cultured for 24 hours in a complete media composed of Dulbecco’s modified Eagle’s medium (DMEM) (Life Technologies) supplemented with 10% (v/v) fetal bovine serum (FBS) (Atlantic Biologicals) and 1% (v/v) antibiotic (penicillin and streptomycin) solution (Life Technologies) under the standard cell culture conditions (a humidified, 37 °C, 5% CO_2_ environment).

### The dose-dependent NO, H_2_O_2_ accumulation in the CAPs media

First, 1 mL of complete media in a well on 6-well plate (Falcon) was treated by CAP for 0.5 min, 1 min, 1.5 min, and 2 min, respectively. The gap between the bottom of the quartz tube and the bottom of plate was 3 cm. 50 μL of CAPs complete media was immediately transferred to a well on a black 96-well clear bottom plate (Falcon) in triplicate. As the control, 50 μL of untreated complete media was also transferred to a well on the same plate in triplicate. Next, according to the standard protocols provided by Promega and Sigma-Aldrich, the NO and H_2_O_2_ concentration in the CAPs media were measured, respectively. The absorbance at 540 nm and the fluorescence at 540/590 nm were read using a H1 microplate reader (Hybrid Technology).

### The liquid surface-dependent NO, H_2_O_2_ accumulation in the CAPs media

First, 1 mL of complete media in a well on 48-well, 24-well, 12-well, and 6-well plate (Falcon) were respectively treated by CAP for 1 min. The gap between the outlet of the quartz tube and the bottom of plate was 3 cm. Then, 50 μL of CAPs media from different multi-well plates were immediately transferred to a well on the black 96-well clear bottom plate in triplicate. For the control, 50 μL of untreated CAPs media was also transferred to a well on the same plate in triplicate. Ultimately, we measured the NO/H_2_O_2_ concentration in the CAPs media.

### The dose-dependent .OH accumulation in the MB solution

The MB solution was prepared by dissolving MB powder into deionized water. Then, 1 mL of 0.01 g/L MB solution in a well on 6-well plate was treated by CAP for 0.5 min, 1 min, 1.5 min, and 2 min. The gap between the outlet of the quartz tube and the bottom of plate was 3 cm. 100 μL of CAPs MB solution was immediately transferred to a well on the black 96-well clear bottom plate in triplicate. As the control, 100 μL of untreated MB solution was also transferred to a well on the same plate in triplicate. Ultimately, we measured the absorbance at 664 nm using a H1 microplate reader (Hybrid Technology).

### The liquid surface-dependent .OH accumulation in the MB solution

First, 1 mL of 0.01 g/L MB solution in a well on 48-well, 24-well, 12-well, and 6-well plate were respectively treated by CAP for 1 min. The gap between the outlet of quartz tube and the bottom of plate was 3 cm. Next, 100 μL of CAPs MB solution was transferred to a well on the black 96-well clear bottom plate in triplicate. 100 μL of untreated MB solution was also transferred to a well on the same plate in triplicate as the control. Ultimately, we measured the absorbance at 664 nm using a H1 microplate reader (Hybrid Technology).

### The dose-dependent anti-cancer capacity of CAPs media

For each cell line, the protocol was identical. Here, we used U87 cells as an example. First, U87 cells were seeded in 96-well plate with three confluencies (2 × 10^4^ cells/ml, 4 × 10^4^ cells/ml, and 8 × 10^4^ cells/ml) and cultured in an incubator for 24 hours under standard conditions. Next, 1 mL of complete media in a well on 6-well plate was respectively treated by CAP for 0.5 min, 1 min, 1.5 min, and 2 min. The gap between the outlet of the quartz tube and the bottom of plate was 3 cm. 100 μL of CAPs media were immediately transferred to culture U87 cells in a well on the 96-well plate in sextuplicate. 100 μL of untreated complete media was also transferred to culture U87 cells in a well on the same plate in sextuplicate as the control. Before this step, the media that was used to culture U87 cells overnight was discarded. After that, U87 cells were cultured in the CAPs media for 72 hours. Ultimately, according to the standard method, the viability of U87 cells were qualified by MTT test and were read by a H1 microplate reader (Hybrid Technology) at the absorbance of 570 nm.

### The well size-dependent anti-cancer capacity of CAPs media

The protocols for three cell lines were identical. Here, we used U87 cells as an example. First, U87 cells were seeded in a 96-well plate with a confluence of 2 × 10^4^ cells/ml and were cultured in an incubator for 24 hours under standard conditions. Next, 1 mL of complete media in a well on 48-well, 24-well, 12-well, and 6-well plate were treated with CAP for 1 min. The gap between the outlet of the quartz tube and the bottom of plate was 3 cm. Then, 100 μL of CAPs media were immediately transferred to culture U87 cells in a well on the 96-well plate in sextuplicate. As the control, 100 μL of untreated media was also transferred to culture U87 cells on the same plate in sextuplicate. Before this step, the media that had been used to culture U87 cells overnight was discarded. After that, U87 cells were cultured in the CAPs media for 72 hour. Ultimately, the cell viability was measured.

### The gap-dependent NO/H_2_O_2_ accumulation in the CAPs media

First, when the gap between the outlet of the quartz tube and the bottom of the plate varied from 2 cm to 4 cm, 1 mL of complete media in a well on 6-well plate was treated by CAP for 1 min. Then, 50 μL of CAPs media was immediately transferred to a well on the black 96-well clear bottom plate in triplicate. 50 μL of untreated complete media was also transferred to a well on the same plate in triplicate as the control. Ultimately, we measured the NO/H_2_O_2_ concentration in the CAPs media.

### The gap-dependent .OH accumulation in the MB solution

First, when the gap between the outlet of the quartz tube and the bottom of the plate varied from 2 cm to 4 cm, 1 mL of 0.01 g/L MB solution in a well on 6-well plate was treated by CAP for 1 min. Next, 100 μL of CAPs MB solution was transferred to a well on the black 96-well clear bottom plate in triplicate. 100 μL of untreated 0.01 g/L MB solution was also transferred to the same plate in triplicate for the control. We measured the absorbance at 664 nm using a H1 microplate reader (Hybrid Technology).

### The gap-dependent anti-cancer capacity of the CAPs media

The protocol for the three cell lines was identical. Here, we used U87 cells as an example. First, U87 cells were seeded in 96-well plate with a confluence of 2 × 10^4^ cells/ml and were cultured in incubator for 24 hours under standard conditions. Next, when the gap between the outlet of the quartz tube and the bottom of plate varied from 2 cm to 4 cm, 1 mL of complete media in a well on a 6-well plate was treated by CAP for 1 min. Then, 100 μL of CAPs media were immediately transferred to culture U87 cells in a well on 96-well plate in sextuplicate. 100 μL of the untreated complete media was also transferred to culture U87 cells in a well on 96-well plate in sextuplicate as the control. Before this step, the media that was used to culture U87 cells overnight was discarded. U87 cells were then cultured in the CAPs media for 72 hours. Ultimately, the cell viability was measured.

### The volume-dependent NO/H_2_O_2_ accumulation in the CAPs media

First, 1 mL, 2 mL, 3 mL, and 4 mL of complete media in a well on 6-well plate were treated by CAP for 1 min. The gap between the outlet of the quartz tube and the bottom of the plate was 3 cm. 50 μL of CAPs complete media was immediately transferred to a well on the black 96-well clear bottom plate in triplicate. 50 μL of the untreated complete media was also transferred to a well on the same plate in triplicate for the control. Ultimately, we measured the NO/H_2_O_2_ concentration in the CAPs media.

### The volume-dependent .OH accumulation in the MB solution

1 mL, 2 mL, 3 mL, and 4 mL of 0.01 g/L MB solution in a well on 6-well plate were treated by CAP for 1 min. The gap between the outlet of the quartz tube and the bottom of the plate was 3 cm. 100 μL of CAPs MB solution was transferred to a well on a black 96-well clear bottom plate in triplicate. 100 μL of untreated 0.01 g/L MB solution was also transferred to a well on the same plate in triplicate for the control. Ultimately, we measured the absorbance at 664 nm using a H1 microplate reader.

### The volume-dependent anti-cancer capacity of the CAPs media

The protocols for three cell lines were identical. Here, U87 cells were used as an example. First, U87 cells were seeded in 96-well plate with a confluence of 2 × 10^4^ cells/ml and were cultured in incubator for about 24 hours under standard conditions. Second, 1 mL, 2 mL, 3 mL, and 4 mL of complete media in a well on 6-well plate were treated by CAP for 1 min. The gap between the outlet of the quartz tube and the bottom of the plate was 3 cm. Then, 100 μL of CAPs complete media was immediately transferred to culture U87 cells in a well on a 96-well plate in sextuplicate. 100 μL of the untreated complete media was also used to culture U87 cells in a well on same plate in sextuplicate for the control. Before this step, the media which has been used to culture U87 cells overnight was discarded. After that, U87 cells were cultured in the CAPs media for 72 hours. Ultimately, the cell viability was measured.

### The anti-cancer capacity of CAPs amino acids rich DMEM

The protocol for three cell lines was identical. Here, U87 cells were used as an example. First, U87 cells were seeded in 96-well plate with a confluence of 2 × 10^4^ cells/ml and were cultured in an incubator for 24 hours under standard conditions. Second, we respectively prepared 2.4 mM specific amino acids rich DMEM by dissolving specific quantities of amino acids powers (Sigma-Aldrich) in DMEM (1% ABS). Because some amino acid rich DMEM would be gradually oxidized by the air during long storage even in the refrigerator, all prepared amino acid rich DMEM were discarded and renewed every two weeks. Next, all 20 specific amino acid rich DMEM and normal DMEM were treated by CAP for 1 min in a well on 6-well plate. The volume of solution in each well was 1 mL. The gap between the outlet of the quartz tube and the bottom of the plate was 3 cm. 100 μL of the CAPs amino acids rich DMEM and the CAPs normal DMEM were transferred to culture U87 cells in a well on 96-well plate in sextuplicate. As the control, 100 μL of the untreated amino acid rich DMEM and normal DMEM were also used to culture U87 cells in a well on 96-well plate in sextuplicate. Before this step, the media which has been used to culture U87 cells overnight was discarded. After that, U87 cells were then cultured in the CAPs media for 72 hours. Ultimately, the cell viability was measured.

### Measurement of NO/H_2_O_2_ in the CAPs amino acids rich DMEM

First, all 20 specific amino acids rich DMEM and normal DMEM were treated by CAP for 1 min in a well on 6-well plate. The volume of solution in each well was 1 mL. The gap between the outlet of the quartz tube and the bottom of the plate was 3 cm. 50 μL of the CAPs amino acid rich DMEM and the CAPs normal DMEM were immediately transferred to a well on the black 96-well clear bottom plate in triplicate. As the control, 50 μL of the untreated amino acid rich DMEM and normal DMEM were also transferred to a well on the same plate in triplicate. Ultimately, we measured the NO/H_2_O_2_ concentration in the CAPs media.

### Measurement of the consumption speed of H_2_O_2_ by cancer cells

The protocol for the three cell lines is identical. Here, we used U87 cells as an example. First, U87 cells were seeded in 96-well plate with a confluence of 1 × 10^4^ cells/ml and cultured in the incubator for 6 hours under the standard conditions. Then, 1 mL of complete media was treated by CAP in 6-well plate for 1 min. After that, 120 μL of the CAPs media was transferred to culture U87 cells on a 96-well plate. Since then, until the third hour, 50 μL of the residual media that was used to culture U87 cells was transferred to a well on the black 96-well clear bottom plate in triplicate. As the control, 100 μL of new complete media was also transferred to a well on the same plate in triplicate. Ultimately, we measured the H_2_O_2_ concentration in the CAPs media.

### The anti-cancer effect of H_2_O_2_ rich media

The protocol for the three cell lines was identical. Here, U87 cells were used as an example. First, U87 cells were seeded in 96-well plate with a confluence of 2 × 10^4^ cells/ml and were cultured in an incubator for 24 hours under standard conditions. Then, 1 μM, 5 μM, 10 μM, 20 μM, 25 μM, 50 μM, and 100 μM H_2_O_2_ rich media was prepared by mixing 30 wt % H_2_O_2_ solution (Sigma-Aldrich) with the complete media. 100 μL of these H_2_O_2_ rich media was transferred to culture U87 cells in a well on 96-well plate in sextuplicate. 100 μL of normal complete media was also used to culture U87 cells in a well of the same plate in sextuplicate for the control. Before this step, the media which was used to culture U87 cells overnight was discarded. After that, U87 cells were then cultured in the incubator for 72 hours. Ultimately, the cell viability was measured.

## Additional Information

**How to cite this article**: Yan, D. *et al.* Principles of using Cold Atmospheric Plasma Stimulated Media for Cancer Treatment. *Sci. Rep.* **5**, 18339; doi: 10.1038/srep18339 (2015).

## Supplementary Material

Supplementary Information
