# Supplementary material for: Principles of using Cold Atmospheric Plasma Stimulated Media for Cancer Treatment
Source: Sci Rep. 2015 Dec 17;5:18339. doi: 10.1038/srep18339 (PMC4683589; doi:10.1038/srep18339)
Supplement: Supplementary Information [file srep18339-s1.doc]

**Supporting materials**

**Principles of using Cold Atmospheric Plasma Stimulated Media for Cancer Treatment**

Dayun Yan^1†^, Annie Talbot^2†^, Niki Nourmohammadi^3^, Xiaoqian Cheng^1^, Jerome Canady^4^, Jonathan Sherman^5^, Michael Keidar^1^*.

^1^Department of Mechanical and Aerospace Engineering, The George Washington University, Science & Engineering Hall, 800 22^nd^ Street, NW, Room 3550, Washington, DC 20052, USA

^2^Columbian College of Arts and Sciences, The George Washington University, Phillips Hall, 801 22^nd^ Street, NW, Suite 212, Washington, DC 20052, USA

^3^Department of Biological Sciences, The George Washington University, Lisner Hall, 2023 G Street, NW, Suite 340, Washington, DC 20052, USA

^4^Jerome Canady Research Institute for Advanced Biological and Technological Sciences, 6930 Carroll Avenue, 3^rd^ floor Suite 300 Takoma Park, Maryland 20912, USA

^5^Neurological Surgery, The George Washington University, Foggy Bottom South Pavilion, 22^nd^ Street, NW, 7^th^ Floor, Washington, DC 20037, USA

† These authors contributed equally to this work.

*e-mail: keidar@gwu.edu


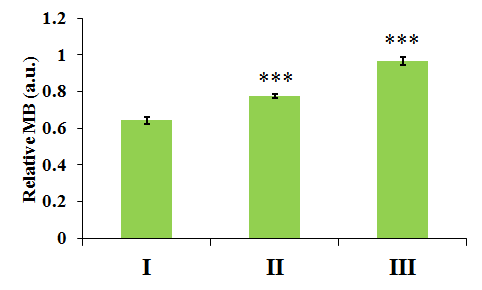


Figure S1. MB solution is sensitive to reactive species with a short half-life. Relative MB absorbance is equal to the ratio between the absorbance of experimental group (I, II, and III) at 664 nm divided by the absorbance of control group at 664 nm. The control group denotes 0.01g/L MB solution. Group I denotes 2 mL 0.01g/L MB solution with 4 min of CAP treatment. Group II denotes 2 mL 0.01g/L MB solution with 2 min of CAP treatment. Group III denotes 2mL deionized water that was first treated by CAP for 4 min. Then, 1 mL of the CAPs water was transferred to mix with 1 mL of untreated 0.02 g/L MB solution. Results are presented as the mean ± s.d. of three repeated experiments performed in sextuplicate. Student’s t-test was performed, and the significance compared with Group I is indicated as * p < 0.05, ** p< 0.01, *** p<0.005.


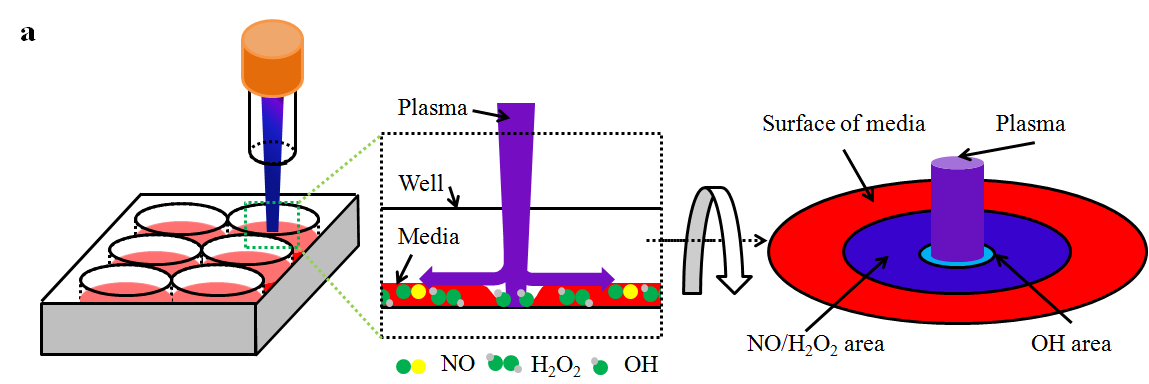


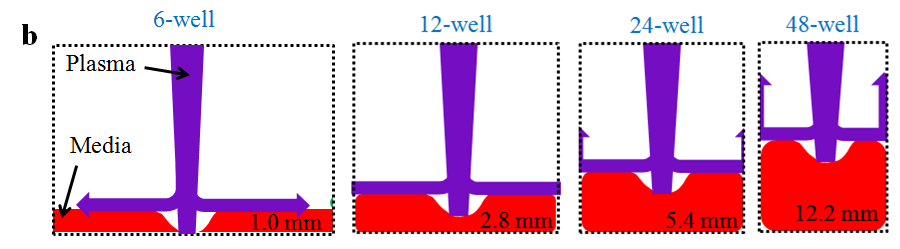


Figure S2. (a) The schematic illustration for the distribution of NO/H_2_O_2_ area and OH area on the surface of media covered by the plasma jet and flow. (b) The mechanism governing the well size-well effect.


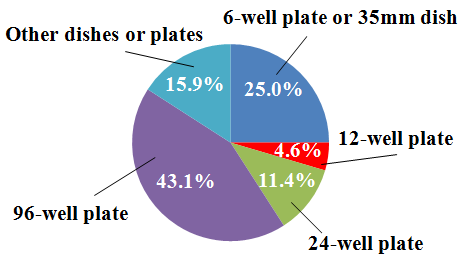


Figure S3. A survey on the multi-well plates or dishes used in research regarding the application of CAP jet in the cancer treatment. The whole number of publication is 44.


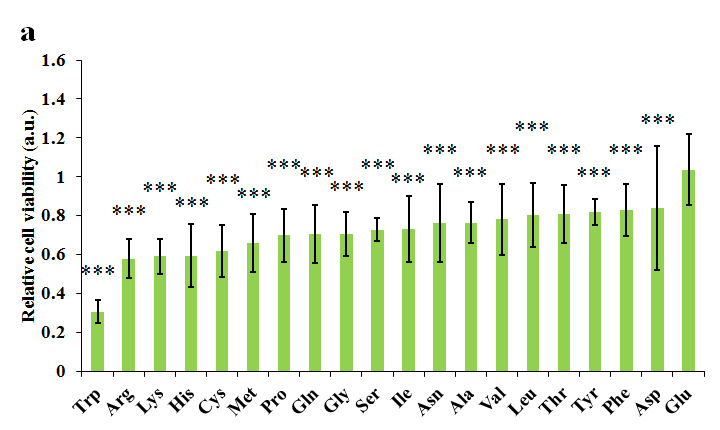


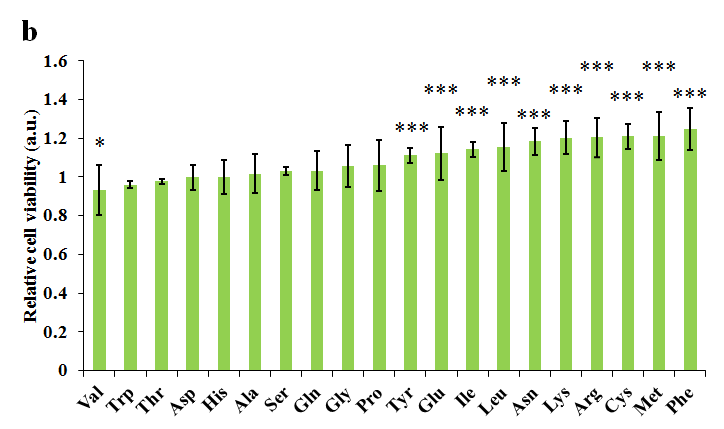


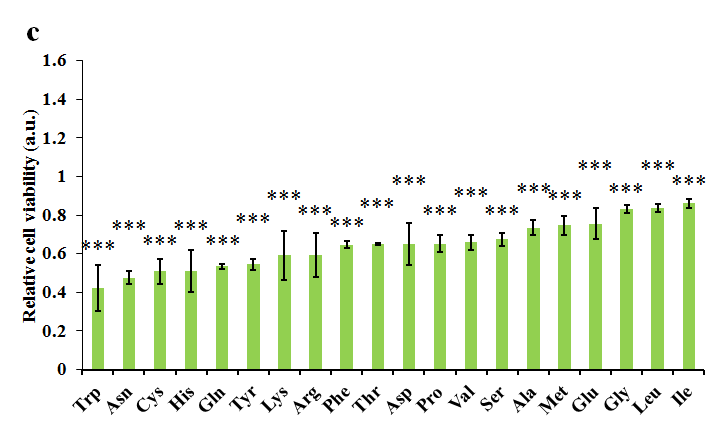


Figure S4. The toxicity of amino acid rich DMEM (2.4 mM) on U87 cells (a), MDA-MB-231 cells (b), and MCF-7 cells. The relative cell viability is equal to the cell viability of cancer cells cultured in the specific amino acid rich DMEM divided by the cell viability of cancer cells cultured in normal DMEM. Results are presented as the mean ± s.d. of three repeated experiments performed in sextuplicate. Student’s t-test was performed, and the significance compared with the cell viability of cancer cells cultured in normal DMEM (not shown in this figure) is indicated as * p < 0.05, ** p< 0.01, *** p<0.005.


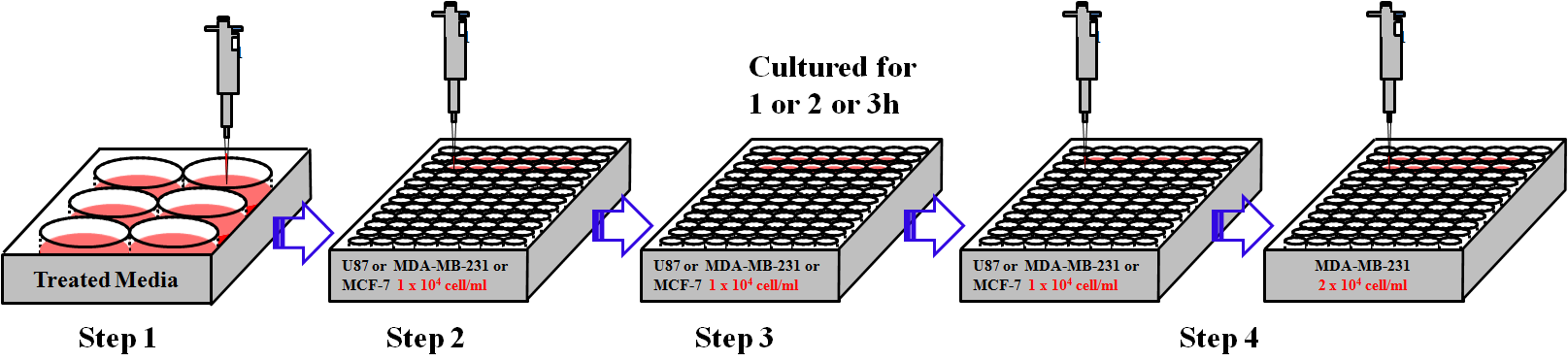


Figure S5. The schematic protocols for using cell probes (MDA-MB-231 cells) to investigate the consumption speed of reactive species by U87 cells, MDA-MB-231 cells, and MCF-7 cells. Before step 1, three cell lines were seeded in 96-well plate with a confluence of 1 x 10^4^ cells/ml and cultured in the incubator for about 6 hours under the standard conditions (not shown in this figure). Step 1: 1 mL of whole media was treated by CAP in 6-well plate for 1 min. Step 2: 120 μL of the CAPs media was transferred to culture three cell lines on a 96-well plate. For convenience, we denoted the residual media to represent the media which had been used to culture three cancer cells. Step 3 and 4: since then, until the third hour, 100 μL of the residual media was transferred from the well to culture cell probes (new MDA-MB-231 cells) in another 96-well plate every hour. These cell probes (MDA-MB-231 cells) had been seed with a confluence of 2 x 10^4^ cells/ml and cultured in the incubator for 24 hours. Simultaneously, as a contrast, 100 μL of CAPs media was immediately transferred to culture cell probes (MDA-MB-231 cells) in the 96-well plate (not shown in this figure). Ultimately, cell probes (MDA-MB-231 cells) in 96-well plates were cultured for 72 hours under the standard culture conditions and experienced cell viability measurement by MTT test.


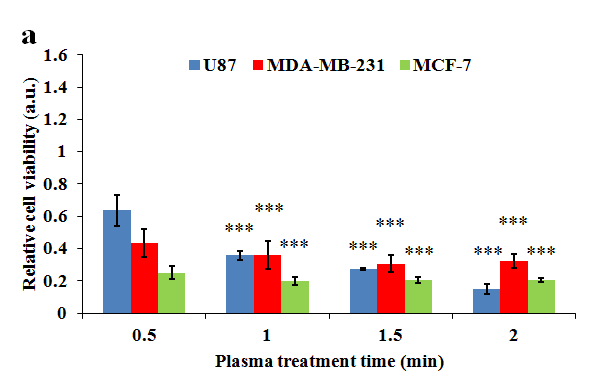


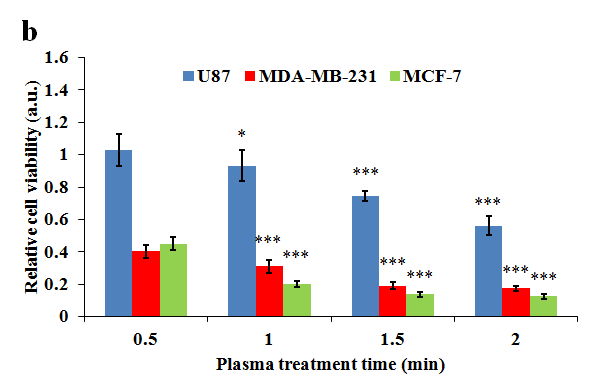


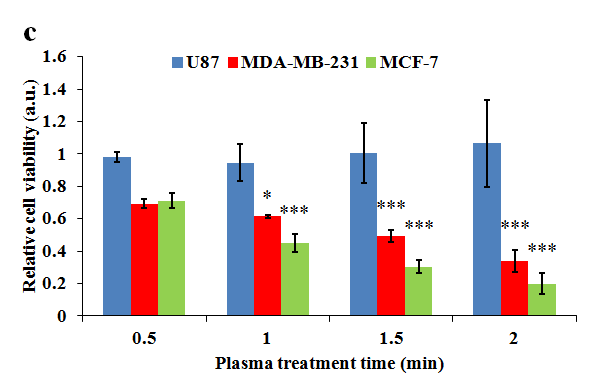


Figure S6. Distinct anti-cancer capacities of the CAPs media on three cancer cells lines with the seeding confluence of 2 x 10^4^ cells/mL (a), 4 x 10^4^ cells/mL (b), and 8 x 10^4^ cells/mL (c). Results are presented as the mean ± s.d. of three repeated experiments performed in sextuplicate. Student’s t-test was performed, and the significance compared with the first bar is indicated as * p < 0.05, ** p< 0.01, *** p<0.005. These data are identical to the data shown in Figure. 4(a), 4(b), and 4(c).
